# Annotations

**Published:** 1897-03-06

**Authors:** 


### Annotations.

### The Greater Cleanliness of the Body.

The Lord Chief Justice of England, better known among the poor of Hackney as Sir Charles Russell, has for many years been a popular person in that eastern suburb; and his words will be treasured in the hearts and minds of many of the sturdy sons of toil long, perhaps, after they have been forgotten by himself. Lord Russell opened a very fine set of public baths on Saturday, baths which have cost no less a sum than £65,000, and which are said to be, in some respects, the finest in England. What chiefly concerns sanitarians in Lord Russell's speech is the statement, well worth recording and thinking about, that in his opinion "greater cleanliness of body is likely to promote greater cleanliness of spirit, and a higher moral tone." It is something for such an utterance as this to come from a lawyer, and so distinguished a lawyer and judge, just as it would be something for it to come from a clergyman so eminent as the Archbishop of Canterbury. We sanitarians are too often told in high legal and ecclesiastical quarters that we "magnify our office"; magnify it, indeed, a great deal too much. Now, we are not at all at one with those who take that view. The body is the basis of everything men and women use and have; it is the physical nidus, if nothing else, of what we call not only "mind" but "soul." The healthy body goes, as a rule, with the healthy mind and soul; and it is almost impossible to find a quite healthy mind and soul apart from a healthy body. In like manner also the clean body, the body that values and makes efforts towards a high degree of cleanliness, is much more likely to value and strive for cleanness of mind and spirit than the body which lives in filth and is content with it. It is one of the problems of a rapidly-growing population how the vast masses are to be kept clean and healthy; and if the poor are not kept clean and healthy the prosperous and the rich have their dangers of illness and disease increased tenfold.

### Railway Nurseries.

The spread of civilisation, while it produces new luxuries for our comfort, also without doubt intrudes upon us new dangers. The last thing in travel is the nursery car. Needless to say we hear of it from America, where the journeys are apt to be so long that it becomes an urgent question what to do with the babes. The travelling nursery is to takes its place alongside of the barber's shop, the bath-room, and other luxuries as yet unknown on English lines, but apparently the recognised appurtenances of the fast trains of America. The walls of this compartment are to be padded, and the floor is to be thickly carpeted, so that bumps and bruises will be avoided. At each end are two little cots, in which the smaller children may lie and watch the games of the older ones. The little travellers will be looked after by a matron or nurse, who will have at hand a supply of milk, cookies, and other edibles and drinkables dear to the infantile heart. She will also, says the *Railway News*, have charge of a medicine chest (!) containing a full assortment of the simpler remedies for childish ailments. A toy shop with everything from picture books to baby rattles is also promised, so that the babies will have a fine time while their mothers loll in the drawing-room car. Medically, however, the suggestion does not seem altogether free from danger. Even as it is, with children but sparsely scattered through a train, there is a definite risk in long journeys of their catching infectious diseases from each other. But for one's child to be shut up with a dozen others of mixed antecedents, not carefully guarded by the watchful care of a suspicious mother, but free to play with all the crowd, and indulge in that community of mugs and goodies which is accepted amongst infants as a mark of mutual confidence, is not altogether an inviting prospect. If there is any infectious disease among them a twelve hours' run in a nursery car ought to give it a fair chance of impartial distribution. The thickly-carpeted floor, the padded walls, the toys, the nurse, the goodies, and even the medicine chest do not seem to the medical mind to counterbalance the risk of promiscuous crowding. Children are a nuisance to other passengers, but there can be no doubt that they are safest on their mothers' knees.

### Therapeutical "Purists."

In a very interesting article in the *Practitioner*, Sir Dyce Duckworth, among other things, points out that therapeutical "purists" are not always the best advisers for the sick. This is especially the case in regard to the prescribing of alcohol in certain diseases, such, for example, as cirrhosis of the liver, which have undoubtedly been caused by alcoholic excess. It is too much the fashion with some to go back to first causes rather than to consider present conditions. True it is that cirrhosis of the liver, the ordinary "hobnailed" gin-drinker's liver, is the result of a long course of alcoholic excess; and so far as the disorder of the liver is concerned probably, even to the end, alcohol is bad for it, and tends to increase the morbid changes which have taken place in that organ. The therapeutical "purist" then would maintain, and might do so with a fair show of logic, that the evil influence which had caused the disease should above all things be withheld. Not so, however, says the wise physician, who looks at his patient from all sides. We have to treat the patient as well as to consider his disease, and when the patient has been much pulled down, when his circulation has failed, so that dropsy has been produced, and perhaps tapping has had to be performed, we must not withhold a stimulant, although we may know that it was the stimulants which brought the sufferer into his miserable plight. "I have noted," says Sir Dyce Duckworth, "that in the later stages patients suffer more miserably if alcohol be withheld from them, pedantically, so to speak, as being harmful to the disease in progress, and that they derive not merely comfort, but benefit from it. Alcohol may be bad for the disease, but it is certainly good for the patient. Pedantry and formality in medicine are, above all things, to be avoided. Our duty is to relieve suffering, even though we may not find it possible to remove the disease from which it springs; and while the medical "purist" may think it contrary to principle to give alcohol in a malady that alcohol has caused, the common sense physician does not hesitate to give a dose of brandy even in such cases, and is often able thereby to afford his patient much relief which could not otherwise have been procured.

